# Materia Medica and Therapeutics

**Published:** 1861-07

**Authors:** Emil Fischer


### MATERIA MEDICA AND THERAPEUTICS; MEDICAL CHEMISTRY; TOXICOLOGY AND LEGAL MEDICINE.

BY EMIL FISCHER, M.D.

### MATERIA MEDICA AND THERAPEUTICS.

I.—*Therapeutical Action of Solanum Dulcamara and Solanine.* By PROF. CAYLUS, of Leipzig.

Professor Caylus has undertaken a series of experiments to ascertain the exact effects of dulcamara, and its active principle, solanine. These substances produce a paralyzing action on the medulla oblongata, and an exciting action on the nerves. They cause death by producing paralysis of the respiratory muscular apparatus, by an action analogous to that of coneine and nicotine. They possess a therapeutical action in spasms and irritable conditions of the respiratory organs in simple spasmodic cough, hooping-cough, and spasmodic asthma. Their therapeutical action in certain morbid conditions of the blood, as gout, rheumatism, constitutional syphilis, and, perhaps, in certain chronic diseases of the skin, may be due to their augmenting the excretion by the kidneys, of the constituent parts of the blood which have undergone combustion, and not to the excitement of cutaneous activity.

Solanine and dulcamara may be given without danger in inflammatory conditions of the stomach and the intestinal tube, as they exercise no action on those organs. Inflammation of the respiratory organs presents no contra-indication to the employment of solanine and dulcamara, but they are contra-indicated in inflammation of the kidneys. The medium dose of solanine for an adult is from one to five centigrammes of acetate of solanine, a substance which M. Caylus prefers to the pure alkaloid, in consequence of its solubility. The most suitable form of administration is in pills, the solution of the salts of solanine having a very disagreeable taste. The extract obtained from alcohol, and then washed with water to remove the alcohol, is preferable to the watery extract generally employed.—(*Med. Times and Gazette*, March 2, 1861.)

II.—*Chuguiragua in Intermittent Fever.* By M. S. ESCOLAR.

According to M. S. Escolar, (*El Siglo Med. Enero*, 1860,) chuguiragua is a shrub-like plant, which grows in Mechoacanna, Mexico, and seems to belong to the natural family of Compositeæ, group of Mutisiaceæ, (*Lessing.*)

The author reports three cases of obstinate intermittent fever in which he used this remedy with excellent success, the disease being cut short either immediately, or after the next paroxysm. He administered the flowers partly in powder, in doses of one scruple daily, suspended in water, partly in the form of infusion, (one drachm to twelve ounces of water,) or an alcoholic extract, in doses of half a scruple daily.—(*Schmidt's Jahrbücher*, 1861, No. 3.)

III.—*Anchieta Salutaris in Chronic Diseases of the Skin.* By M. TH. PECKOLT.

Anchieta salutaris, a creeping shrub, is a native of Brazil, where, according to the author, the bark of its root is considered an efficacious remedy in chronic affections of the skin. The powder of the bark of the root is given in doses of six to twelve grains daily, and its administration continued for one and a half to two months, the dose being increased every week by an additional grain. In the commencement it acts as a drastic purgative, but, after three or four days, causes not more than one or two evacuations a day.

The root is much furrowed; the inner woody portion covered with a thick, porous, and juicy bark, which is easily peeled off. The cortical substance is reddish brown externally, and flesh colored internally. The pith of the root consists of firm, tasteless, ligneous fibres; iodine colors the cut surface of the bark violet, the pith brownish. M. Peckolt found in the bark of the root albumen, gum, starchy substance, sugar, gluten, resin, soluble in ether, and a peculiar crystallizable body—anchietin. From two pounds of the bark sixty-three grains of irregular, needle-shaped, straw-colored crystals are obtained. Anchietin has a nauseous, pungent taste, is inodorous, insoluble in ether and cold water, and slightly soluble in hot water; in alcohol it dissolves abundantly; its solution is of slightly alkaline reaction. On a plate of platinum it becomes almost completely volatilized, under formation of thick fumes. It neutralizes diluted acids, and forms with them partly crystallizable salts; the combination with muriatic acid, among others, presents itself under the shape of beautiful, white needles, grouped in the form of stars; these are redissolved in boiling water, but are nearly insoluble when dried. From the solution ammonia precipitates the straw-colored anchietin. On treating anchietin with concentrated nitric acid, it assumes at first a beautiful orange-yellow, and afterward a chrome-yellow color, without being dissolved; by concentrated sulphuric acid it is colored at first violet, then blackish; concentrated muriatic acid renders its original yellow color more intense.—(*Prager Vierteljahrschrift*, 1861, vol. i., Analekten.)

IV.—*Phosphate of Ammonia in Rheumatism.* By DR. HAMOLECKI.

The author recommends phosphate of ammonia in the treatment of rheumatism. According to Bergson, this remedy was brought into notice by Dr. Buckler, an American physician, who maintained that the uric acid diathesis and sediments disappear after its use. M. Edwards sustained this statement, believing that the formation of an abnormal quantity of uric acid in the blood was the principal cause of rheumatism and gout, and that phospate of ammonia formed with the uric acid and urate of soda in the body two easily soluble salts, phosphate of soda and urate of ammonia. M. Delioux fears the formation of calculi of phosphate of ammonia and magnesia, and does not consider it safe to administer nitrogenous substances, such as ammonia, in rheumatism. M. Rieken, of Brussels, has given phosphate of ammonia in rheumatism without success, but recommends the use of benzoate of soda. Dr. Hamolecki tried the methods of Bouillaud, (venesections,) of Laennec, (tartar emetic,) and of Martin Solon, (nitrate of potassa,) in vain, and, in a desperate case, finally had recourse to phosphate of ammonia, having had his attention drawn to this remedy by Dr. Mattei. His success in this and all other cases was so favorable that, in his practice in the hospital of Kremienczug, he has had, for the last four years, no reason to adopt any other method of treatment. He administers the salt to the amount of two drachms daily.—(*Deutsche Klinik*, and *Prager Vierteljahrschrift*, 1861, vol. i., Analekten.)

V.—*Therapeutic Studies on Essence of Valerian.* By M. BARAILLER.

The following conclusions have been attained by Barailler:—

1. Employed in the case of a healthy man, the essence gives rise to several symptoms, the principal of which are, intellectual sluggishness, drowsiness, deep sleep, reduction in number of arterial pulsations, and, later, an increase and abundant flow of urine.

2. Given to a sick man, it modifies, in a prompt and rapid manner, the symptoms, stupor, somnolence, coma from some dynamic cause, which complicate severe fevers.

3. This modification is obtained by administering from 50 centigrammes to 1 gramme (from 10 to 20 drops) of the essence in twenty-four hours.

4. The action of this remedy can only be explained by the employment of the law of similitude, enunciated by Hippocrates, and by a number of the ancient authors.

5. Certain nervous conditions, such as vertigo, hysteria, essential asthma, etc.

are modified in a remarkable manner by the volatile oil of valerian, which, under the results of a series of new experiments, is capable of extending the sphere of therapeutic applications of this plant very much.—(*Jour. and Trans. Md. Col. Pharm.*, March, 1861; from *Répertoire de Pharm.*)

VI.—*A New Salt of Iron and Quinine.* (Communicated by Dr. Fergus, of Marlborough College, to the Medical Times and Gazette, March 17th, 1861.)

It is generally found that a salt of the protoxide of iron is preferable to one of a higher degree of oxygenation; but it is also difficult to obtain an absolutely permanent salt of the protoxide. Perhaps, without exception, the sulphate is the most practically useful of all the salts of iron, owing to the uniformity of its composition. Of the quinine salts, the sulphate is also the most available for general purposes. It is not difficult to form a simple combination of these two sulphates, but the resulting compound is not well fitted for general use. The addition, however, of a certain proportion of sulphate of magnesia enables us to obtain a salt which is nearly as soluble as the sulphate of magnesia itself, quite unalterable in the solid state, and forming a solution perfectly clear at first, and remaining so for an indefinite period. The iron has no tendency to a further state of oxygenation; the solution has been agitated with oxygen gas, and kept in contact with it for several days, without the least change. A solution of gallic acid tinges a solution of the salt a light bluish color, after the lapse of two or three days, and many substances which produce an inky compound with the salts of iron may be mixed with it without causing any change of color.

The proportion of the three sulphates which has been adopted, is 80 per cent. of sulphate of magnesia, 15 per cent. of sulphate of iron, and 5 per cent. of sulphate of quinine, one scruple containing 16, 3, and 1 grains of the respective salts. These proportions have been found the best for general use, and also for the purposes of manufacture. The proportion of quinine may be increased by prescribing an additional quantity which is readily soluble in the solution of the salt.

One peculiarity is especially deserving of notice: that in this combination the assisting or adjuvant property of both iron and quinine are remarkably developed, the effect of both, particularly of quinine, being heightened in a very marked manner. At the same time, both of the remedies are less apt to disagree with peculiar constitutions which ordinarily refuse to tolerate either iron or quinine. If the heightened power be borne in mind in prescribing this combination, there will be very few cases found in which it will not be suitable whenever either iron or quinine are indicated.—(*London Pharm. Journ.*, April, 1861.)

VII.—*Coal-Tar Emulsion.* By M. Demeaux.

M. Demeaux, in a note to the Academy, recommends the following preparation of coal-tar—a substance much used now in the French hospitals as a disinfectant. This preparation, he says, is recommended by the facility with which it is made, the quantity of coal-tar it contains, and its great solubility. Of coal-tar, soap, and alcohol, equal parts are to be heated in a bath until complete solution is effected, and, on cooling, a very soluble soap is produced, which, with water, makes a stable emulsion. It costs about a franc the kilogramme, and three kilogrammes will emulsify 100 litres of water, each litre containing ten grammes of coal-tar; and in this form may be very advantageously used in hospitals and dissecting-rooms, in certain manufactures, and other places where insalubrious inhalations prevail. The coal-tar soap thus combined is a very manageable substance, and may be used in any desired degree of concentration, and either in the solid or fluid form—its great solubility in cold or warm water preventing its soiling any substance it comes in contact with. It may be usefully employed in baths in some diseases of the skin; while, as a dressing, it is of good service in cases in which the excretions give rise to fetid emanations.—(*Bull. de Thérap.*, vol. lx. p. 130, and *Med. Times and Gaz.*, March 23, 1861.)

VIII.—*On Sulphurous Powder.* By MARCELLIN POUILLET.

The formula of the Codex for the preparation of artificial sulphurous waters for drinking is inefficient and badly studied. The Academy of Medicine, on the report of M. Robinet, has adopted the following formula, due to the researches of M. Marcellin Pouillet, which attains the objects of a good preparation, keeping perfectly well and being economical:—

| | |
|---|---|
| Sulphuret of Calcium, | Sulphate of Potassa, |
| Bicarbonate of Soda, | Gum-arabic, |
| Sulphate of Soda, | Tartaric Acid. |

Equal parts of these substances, well dried, are reduced to powder and mixed.

Fifty centigrammes ($7\frac{3}{4}$ grs.) dissolved cold in a litre ($2\frac{1}{9}$th pints) of water gives, after standing a quarter of an hour, a sulphurous water, which it is impossible to distinguish from the natural sulphurous waters.

The reaction which occurs between the different elements of this powder is easily understood. Tartaric acid and bicarbonate of soda produce carbonic acid; and this acid, in the presence of sulphuret of calcium, gives rise to a disengagement of sulphuretted hydrogen gas, which is dissolved in the liquid. As all the sulphuret of calcium is decomposed, it results that the water is always identical when that compound is pure, and this is a condition essential to a good preparation.

The trials made by MM. Bazin, Cazenave, and Richet have given the most satisfactory results, and M. Bouchardat himself has prescribed with advantage this sulphurous powder.—(*Répertoire de Pharm.*, Feb., 1861, and *Am. Journ. of Pharmacy*, May, 1861.)

IX.—*Æmilia Rigidula De C.; a Tonic and Antichlorotic from Guyana.*

Mr. Michely, of Cayenne, has sent to the *Société d'Acclimatation* some seeds of this plant of Guyana, which is a very powerful tonic and deserves to be ranked among the best bitters of the materia medica. The plant is cultivated the same as bitter chicory, and is taken in the form of salad, with vinegar, salt, and oil, an hour before meals or in the morning. At the same time a tea is taken prepared from the flower-tops. Some remarkable cases are on record, and the firm of Vilmorin, Andrieux & Co., of Paris, are now cultivating the plant for the sake of general introduction.—(*Am. Druggists' Circular*, May, 1861.)

### X.—*Chlorate of Potash and Glycerin as a Topical Disinfectant.*

Experiments instituted at Bicêtre, under the direction of M. Martinet, have demonstrated remarkable disinfecting properties in a mixture of chlorate of potash and glycerin according to the following formula: Chlorate of potash, in powder, 2½ drachms; glycerin, 3 ounces; mix. This mixture has been shown, by repeated trials, to present—1. A marked disinfecting power, due perhaps to the change which it produces in the secretion, and the mode of action of the wound. 2. The property of giving the pus, even when of a serous kind, a greater consistence, often like cream. This result is perhaps, according to M. Martinet, a physical effect of the affinity of glycerin for water, which it subtracts from the pus; but is partly due to the favorable modification which is produced in the suppurating surface. He is inclined to think that the preparation of glycerin and chlorate of potash may, by thickening the pus, tend to prevent the occurrence of purulent or putrid infection, which generally takes place in connection with suppuration of a serous and unhealthy character. An advantage of the glycerin is, that it prevents the dressings from sticking to the edges of wounds. According to M. Martinet, the glycerin chlorate of potash is not adapted for wounds or sores of a bright red color, nor for those that are recent or of healthy appearance.—(*Rép. de Pharm.*, and *Dub. Med. Press*, April, 1861.)

### XI.—*Ailanthus Glandulosa as Anthelmintic.* By DR. HÉTET.

From an article contained in the Journal *l'Institut* of February 8, 1860, and republished in *lo Sperimentale*, a Florence journal, (July, August, and September, 1860,) it results that our list of anthelmintics is to be augmented by one more remedy, said to be useful especially in cases of tænia. Dr. Hétet, Professor at the School for Naval Medicine in Toulon, has ascertained, by experiments made on animals, and repeated afterward on man, that the *Ailanthus glandulosa*, of the family of *terebinthaceæ*, possesses decided anthelmintic properties. This tree, coming originally from China, is at present much cultivated in Europe as an ornament, and also on account of the utility of its wood and leaves, which serve a particular species of silk-worm as nourishment. The anthelmintic property resides as well in the leaves as in the bark; reduced to powder and given in doses increasing from 50 centigrammes to 2 grammes, they expel the tænia without the least injury to health, and without producing the nausea caused by the bark of pomegranate root and by kousso, the only unpleasant effect being some griping and sometimes slight purgation.

Since the first experiments of M. Hétet, this remedy has been employed as vermifuge on the vessels of the French navy, and always with success. Ailanthus may thus be added to the other anthelmintics, over which it has the advantage of being borne by the system without inconvenience, and of being found everywhere.—(*Journal de Médecine de Bordeaux*, December, 1860.)

### XII.—*On the Therapeutical Properties of Bromide of Potassium.*

According to the researches of M. Huette, the administration of the bromide of potassium produces prostration of strength, difficulty in moving, diminution of general sensibility, anæsthesia of the mucous membrane of the palate and larynx, and more or less torpor of the genital organs. M. Thielmann has employed this salt successfully in the treatment of painful erections, satyriasis, and spermatorrhœa. Dr. Pfeiffer has obtained the same results, and has used the bromide in abnormal erections, neuralgia of the neck of the bladder, catarrh of the urinary passages, and gravel. The efficacy of the mineral water of Kreuznach, in the latter affections, is due to the large quantity of bromide of potassium and magnesium contained in this water. M. Pfeiffer administers the bromide of potassium in the dose of 50 centigrammes, raising it progressively to 2 or 3 grammes, (℈ss. to three-fourths of a drachm;) he gives it in two doses, night and morning, or in doses otherwise divided to suit particular constitutions. —(*Bulletin Général de Thérapeutique*, and *Druggists' Circular*, March, 1861.)

---

### Toxicology and Legal Medicine.

### I.—*Hœmatine as a Test in Forensic Medicine.*

Hæmine and Hæmatine do not present as yet the slightest pathological interest; but, on the other hand, they have proved of very great importance in forensic medicine, on account of their having been recently employed as one of the surest tests for the examination of blood stains. I myself have been in a position to make experiments of this sort in forensic cases. For this purpose, the best mode of proceeding is to mix dried blood in as compact a form as possible with dry, crystallized, powdered common salt, and then to add to this mixture glacial acetic acid, and evaporate at a boiling heat. When this has been done crystals of hæmine are found where the blood-corpuscles or the substance previously lay, in which the presence of hæmatine was doubtful. This is a reaction which must be ranked among the most certain and reliable ones with which we are acquainted. There is no other substance in which we know such a transformation to take place but hæmatine. This test is extremely important, because it is applicable in the case of extremely minute quantities, only they must not be spread over too large a surface. It would, therefore, not be easy of application in a case where we had to deal with a cloth which had been dipped into a thin watery fluid colored with blood. Yet I was able, in the case of a murdered man, on the sleeve of whose coat blood had spirted, and where some of the drops were only a line in diameter, from these minute specks to produce innumerable crystals of hæmine, though of course microscopical ones. In cases in which the ordinary chemical tests would necessarily absolutely fail, on account of the smallness of the quantity, we are still able to obtain hæmine.—(*Virchow's Cellular Pathology; Medical Times and Gazette*, February, 1861.)

### II.—*Poisoning by the Arum, or Cuckoo-pint,* (*Arum maculatum of Linnœus.*) By M. Cangella.

A child three years of age, having masticated, on the 20th of April, 1860, at 2 p.m., some roots of the Arum, commonly called cuckoo-pint, complained immediately of burning pains in the mouth and lips. Three hours afterward it was in a state of profound torpor, to which succeeded a violent febrile reaction.

At eight o'clock I found the little patient in a prostrate condition, unable to speak, raising its hands frequently to its mouth and throat, and uttering at intervals a sharp cry, and starting up as if suffocated. I observed that the caustic action of the plant had extended to the lips, the palate, the tongue, the tonsils, the pharynx, and wherever I could see. The pain of the stomach, on pressing it, indicated to me that the child had swallowed the juice, and that its caustic action had extended thus far.

Not having any means at hand to produce the evacuation of the poisonous matter, I administered a solution of common salt. But deglutition was impossible; the swelling was such that I could not even introduce a probang into the œsophagus. The most energetic revulsive agents were employed without success, and death took place at eleven o'clock, during delirium, from asphyxia.—(*Gazetta Medica do Porto*, and *London Pharmaceutical Journal*, February, 1861.)

III.—*Euphorbia Lathyris as a Poison.*

Three workmen of Toulouse, engaged in the tobacco manufacture, conceived and carried out lately the singular notion of purging themselves immediately after breakfast. They selected for this purpose the seeds of *Euphorbia Lathyris*, which are very much used as a purgative agent among the peasants of that country. One workman took eighteen seeds, and the other two twenty and twenty-five respectively.

A short time after the ingestion of the seeds, they produced violent purgation, vomiting, frequent diarrhœa, and a general pallidness and coldness spread over the body. These symptoms, which strikingly resemble those produced by an attack of sporadic cholera, yielded to appropriate treatment, and in the evening of the same day the three imprudent workmen had entirely recovered.

This case confirms what has already been experimentally proved by Dr. Augustus Dassier, namely, that the seeds of certain euphorbiaceous plants, as those of *Ricinus communis*, when swallowed whole, exert a far more violent action than that of the oil extracted from the same seeds.

According to Sorbeiran, the seeds of the *Euphorbia Lathyris* contain an acrid matter, which is only partly soluble in the yellow fixed oil which they also contain. Hence we have another fact which serves to corroborate and explain the difference before noticed in the mode of action of the oil and the entire seeds.—(*Répert. de Pharm.*, and *London Pharm. Journal*, February, 1861.)

IV.—*Poisonous Effects of Euphorbia Peplus.*

The following case, recently communicated to the Academy of Sciences by Dr. D. Bernard, will also illustrate the disastrous effects which may result from the empiric employment of the indigenous euphorbiaceous plants:—

A lady, following the advice of an old woman, cut some stems of *Euphorbia Peplus*, and applied the milky juice flowing from them to her already swollen gums. Instantly her mouth became so much inflamed that the doctor, when he saw his patient, thought that the inflammation was due to the action of mercurial medicines.

For several days the pain was intolerable, and wrung cries from the patient, who, deprived of sleep and unable to drink, was a prey to indescribable agitation.

By the aid of lotions combined with emollient and opiate injections, and by the employment of revulsives and laudanumized poultices, and assisted especially by time, the action of the caustic became weaker, the inflammation gradually diminished, and at the end of eight days the cure was nearly complete.—(*Ibidem.*)

V.—*Poisoning by the Berries of Solanum Dulcamara.*

While we are upon poisonous agents, we may notice a fatal case of poisoning which occurred lately near Toulouse. It happened to a child who had swallowed some berries of *Solanum Dulcamara*, and who, notwithstanding the greatest care which was given to it, died the same day.

This fact is important, as some authors—among others M. Guibourt—have proved that the plants of the genus *Solanum Dulcamara* possess far less poisonous properties than those of the genera Datura and Atropa, which belong to the same natural order.—(*Ibidem.*)

VI.—*Arsenical Poisoning.*

A remarkable case of poisoning by arsenic was tried at the Durham Assizes, in which it was proved that the accused person, the wife of the deceased, purchased a quarter of a pound of arsenic, ostensibly to cure her husband's toothache. It was sold to her labeled mercury. He died shortly after taking some medicine which she administered, exhibiting symptoms of arsenical poisoning. In his stomach was found one hundred and fifty grains of arsenic. It was proved, on the one hand, that she had contemplated her husband's death; but it was proved also, on the other, that the deceased was in the habit of using the "mercury," that is to say, the arsenic, for his teeth, and that she had seen him put a quantity of it into a lucifer box, and take a spoonful from it, into which he had dipped his finger, when he had wetted it, and rubbed it into his gums. The teeth of the deceased were proved to be very much decayed, but no irritation of the gum, or sign of the recent application of arsenic, appeared. Of course no conviction could follow after such evidence.—(*Dublin Medical Press*, and *Journ. of Materia Med.*, February, 1861.)

VII.—*Poisoning by Cyanide of Potassium.*

F. Venghauss obtained from the blood of a man, who had died from accidentally drinking a solution of cyanide of potassium, free hydrocyanic acid, but found none therein in combination. The stomach was free from the free acid, a mixture of protosulphate and sesquichloride of iron having been given as an antidote, too late, however, to save life.—(*Arch. d. Ph.*, clii., 138, 142, and *Am. Journ. of Pharm.*, May, 1861.)

